# Suspected Feline Calicivirus Infection Triggering Ulcerative Oral and Skin Lesions in Cats Following Routine Ovariohysterectomy: A Postoperative Risk Assessment

**DOI:** 10.1002/vms3.70540

**Published:** 2025-07-29

**Authors:** Ebru Karakaya‐Bilen, Gülşah Akgül, Oznur Yılmaz‐Koc

**Affiliations:** ^1^ Department of Obstetrics and Gynecology, Faculty of Ceyhan Veterinary Medicine Çukurova University Adana Türkiye; ^2^ Department of Internal Medicine, Faculty of Veterinary Medicine Siirt University Siirt Türkiye; ^3^ Department of Obstetrics and Gynecology, Faculty of Veterinary Medicine Siirt University Siirt Türkiye

**Keywords:** caliciviridae, cat, ovariohysterectomy, oral and skin lesions

## Abstract

Feline calicivirus (FCV) is a highly contagious pathogen prevalent in domestic cats, often leading to upper respiratory tract infections and oral diseases. In clinical settings, particularly veterinary hospitals and shelters, nosocomial outbreaks of FCV can occur, sometimes involving virulent systemic disease (VSD) strains that result in severe systemic illness with high mortality rates. This retrospective study reviews the clinical and diagnostic features of 15 cats that developed postoperative ulcerative tongue lesions, ptyalism and skin lesions after elective ovariohysterectomy (OVH) performed at a veterinary teaching hospital. In all documented cases, the initial postoperative clinical manifestation was the development of widespread ulcerative lesions on the tongue, accompanied by ptyalism. Accompanying signs included anorexia, lethargy, fever, dysphagia and various dermatological manifestations. Although healing took longer for those with skin issues, recovery was achieved in all cases. While some clinical features resembled virulent systemic FCV (VSD‐FCV), no multi‐organ dysfunction or mortality occurred, and the diagnosis remained presumptive due to the absence of molecular confirmation. Instead, diagnosis of suspected FCV infection was made based on consistent clinical signs and positive antibody detection using a fluorescent immunoassay rapid test. Cats were primarily treated with antibiotics, nonsteroidal anti‐inflammatories and corticosteroids; however, in some cases, extended treatment was provided based on the observed symptoms. The clinical and serological findings suggest that early recognition of suspected FCV cases and immediate intervention can contribute to favourable outcomes and help limit potential nosocomial spread. To the authors’ knowledge, this is the first report from Turkey documenting suspected FCV clusters following OVH, offering observational insight into potential postoperative transmission.

In conclusion, although OVH is a standard procedure, it is crucial to implement stringent infection control measures to prevent the spread of FCV and other infectious agents in veterinary settings.

## Graphical abstract

This study describes suspected feline calicivirus (FCV) infections occurring after elective ovariohysterectomy in cats at a veterinary hospital in Turkey. Clinical signs, diagnostic approach and response to treatment were evaluated, highlighting the importance of early detection, supportive care and strict infection control to prevent nosocomial outbreaks.

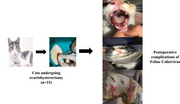

## 1 Introduction

Feline calicivirus (FCV) is a highly contagious virus belonging to the Caliciviridae family, responsible for upper respiratory infections, oral ulcerations and, in severe cases, virulent systemic disease (FCV‐VSD) in cats (Radford et al. 2007; Gaskell et al. 2006). FCV utilizes feline junctional adhesion molecule A (fJAM‐A) as its primary cellular receptor, which is located at the tight junctions of endothelial and epithelial cells (Hofmann‐Lehmann et al. 2022). Disruption of these tight junctions by FCV infection compromises the epithelial barrier, leading to oral ulceration in the classic form of the disease and cutaneous ulceration in the virulent systemic form. The ability of FCV to target fJAM‐A is a key factor in its pathogenesis, influencing both local and systemic disease manifestations. FCV exhibits significant genetic and antigenic variability, contributing to differences in clinical presentation and disease severity (Reynolds et al. 2009; Bordicchia et al. 2021; Park et al. 2024). It appears that FCV vaccination reduces the severity of classical calicivirosis, but fails to protect animals from infection (Radford et al. 2009; Bordicchia et al. 2021) or the systemic form (Radford et al. 2007).

Elective surgical sterilization of female cats is one of the most commonly performed procedures in veterinary practice. In addition to the potential benefits of this surgical procedure, there are some risks that can be seen during or after the operation. In general, these risks can be bleeding, complications related to general anaesthesia, delayed healing at the operation site, hernia, abscess or other unpredictable problems (Roberts et al. 2015; Kreisler et al. 2018).

Nosocomial infections, or hospital‐acquired infections, are a critical concern in veterinary medicine, as they can facilitate the rapid spread of highly virulent pathogens, including FCV. Infected cats may shed the virus through respiratory secretions, saliva and fomites, increasing the risk of cross‐contamination among hospitalized patients. Inadequate biosecurity measures, improper disinfection protocols and the presence of asymptomatic carriers further contribute to the emergence of nosocomial outbreaks (Hurley et al. 2004; Deschamps et al. 2015). Nosocomial FCV‐VSD outbreaks have been reported in the United States, Europe and other countries. FCV‐VSD outbreaks with high overall mortality rates have been reported (Schorr‐Evans et al. 2003; Hurley et al. 2004; Deschamps et al. 2015; Caringella et al. 2019; Park et al. 2024).

This study presents the first reported cluster of suspected FCV cases in eastern Turkey, describing the clinical characteristics, possible risk factors, presumptive diagnostic findings and treatment outcomes observed in cats following elective ovariohysterectomy.

## 2 Materials and Methods

### 2.1 Data Collection and Clinical Observations

**FIGURE 1:**
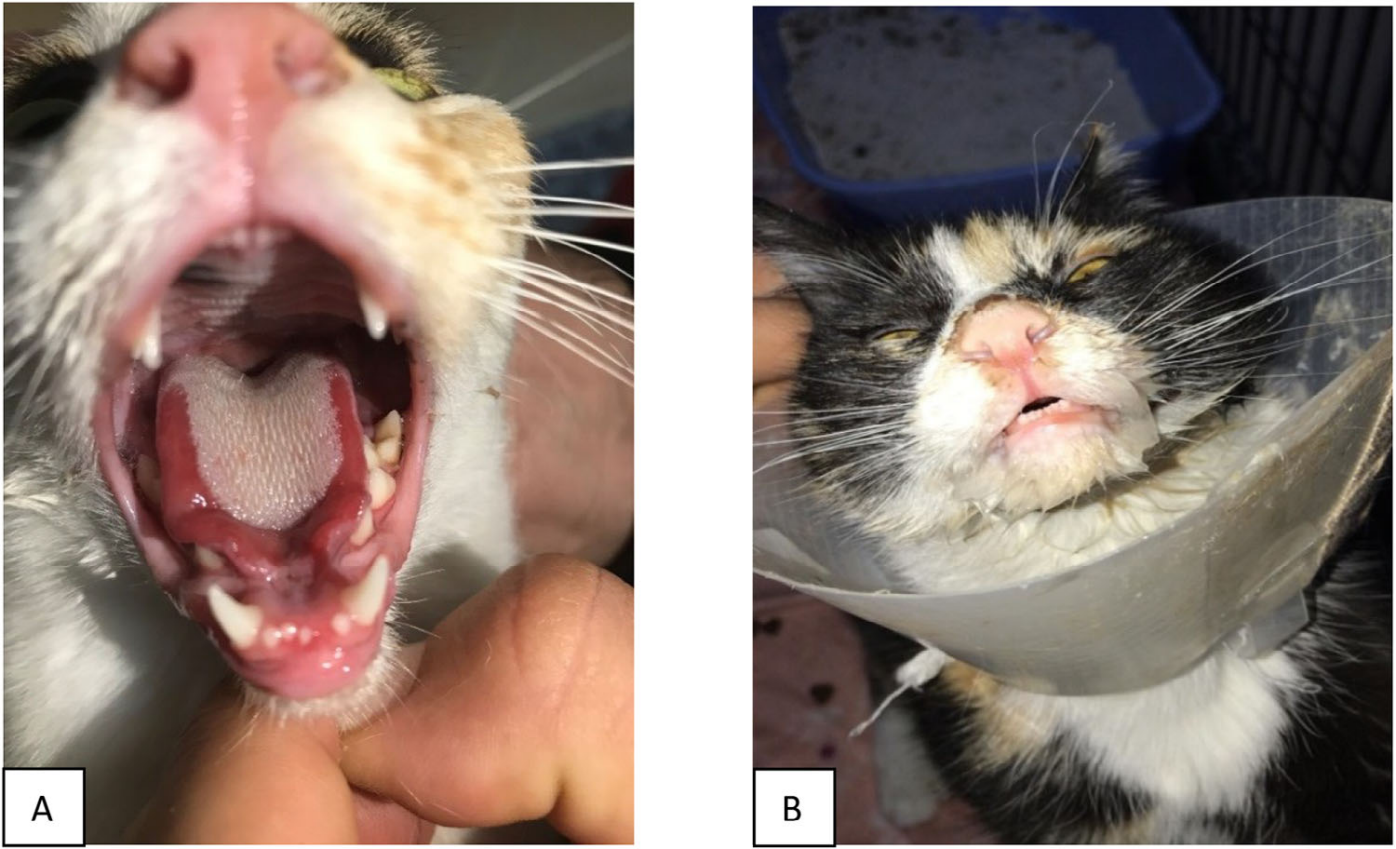
Tongue ulcer (A) and hypersalivation (B) in cats with feline calicivirus infection.

Routine ovariohysterectomy was performed on 15 cats of different breeds weighing an average of 3 kg, which were found to be clinically healthy as a result of general and laboratory examinations before OVH. According to the veterinarian's preference, the operation was performed on a median or lateral approach. Thirteen of the cats were owned, while two were strays, and their mean age was 1.5 years. Eight of the cats were purebreds, while seven were domestic shorthairs. In addition, vaccinations and parasite treatments were completed for seven of these cats. Three clusters of suspected FCV infection were observed during the year, each involving cats developing similar clinical signs following ovariohysterectomy in the same clinical setting. Data were collected after routine OVH between 2021 and 2022; the first symptoms seen in all cats within a few days after surgery were the development of oral lesions on the tongue with hypersalivation (Figure 1). Medical records were reviewed to document clinical signs, duration of symptoms, laboratory findings and response to treatment (Table 1). Data collected included signalment, medical history, vaccination status and intraoperative conditions. The average time for the first appearance of these symptoms was recorded as 50.9 h, with a minimum of 2 h and a maximum of 120 h. In addition to hypersalivation, dysphagia was observed in nine cats, and ulcerative dermatitis was noted in six cats, with an average duration of three days. In addition to clinical findings similar to those seen in VSD associated with FCV infection, antibody titre measurements were performed with the Fluorescent Immunoassay Rapid test (Finecare, Wondfo Biotech Co. Limited, Finecare, Atateknik, Turkey). Also, samples were collected as oral and laryngeal swabs and blood to be submitted to the veterinary diagnostic laboratory.

**TABLE 1:** Of the cats, whose average age was 1.5, 13 were owned and two were stray. It was reported that seven of these cats were fully vaccinated and treated for parasites.

Case no.	Owned	Vaccination status	Breed	Duration of clinical symptoms (hours)	İnitial clinical complaint	Lesion area	Skin lesion onset (days)	Average recovery time (days)
**1**	Yes	Unvaccinated	Street	72	Lesion on tongue, hypersalivation, anorexia	Tongue	—	4
**2**	Yes	Unvaccinated	Street	12	Lesion on tongue, hypersalivation, anorexia	Tongue	—	7
**3**	Yes	Vaccinated	Scottish Fold	24	Lesion on tongue, hypersalivation, anorexia, skin lesion	Tongue, skin	4	7
**4**	Yes	Vaccinated	Scottish Fold	24	Lesion on tongue, hypersalivation	Tongue, skin	2	2
**5**	No	Unvaccinated	Street	24	Lesion on tongue, hypersalivation	Tongue	—	4
**6**	Yes	Unvaccinated	Van	36	Lesion on tongue, hypersalivation, anorexia, skin lesion	Tongue, skin	2	5
**7**	Yes	Unvaccinated	Russian Blue	18	Lesion on tongue, hypersalivation, anorexia, skin lesion	Tongue, skin	4	5
**8**	Yes	Unvaccinated	Street	24	Lesion on tongue, hypersalivation, anorexia, skin lesion	Tongue, skin	2	5
**9**	No	Unvaccinated	Street	120	Lesion on tongue, hypersalivation, anorexia, skin lesion	Tongue, skin	5	7
**10**	Yes	Vaccinated	Scottish Fold	96	Lesion on tongue, hypersalivation	Tongue	—	5
**11**	Yes	Vaccinated	Scottish Fold	24	Lesion on tongue, hypersalivation	Tongue	—	3
**12**	Yes	Unvaccinated	Street	120	Lesion on tongue, hypersalivation, anorexia, skin lesion	Tongue, skin	5	5
**13**	Yes	Vaccinated	British Shorthair	96	Lesion on tongue, hypersalivation, anorexia	Tongue	—	5
**14**	Yes	Vaccinated	Street	72	Lesion on tongue, hypersalivation	Tongue	—	3
**15**	Yes	Vaccinated	Scottish Fold	2	Lesion on tongue, hypersalivation	Tongue	—	5

**FIGURE 2:**
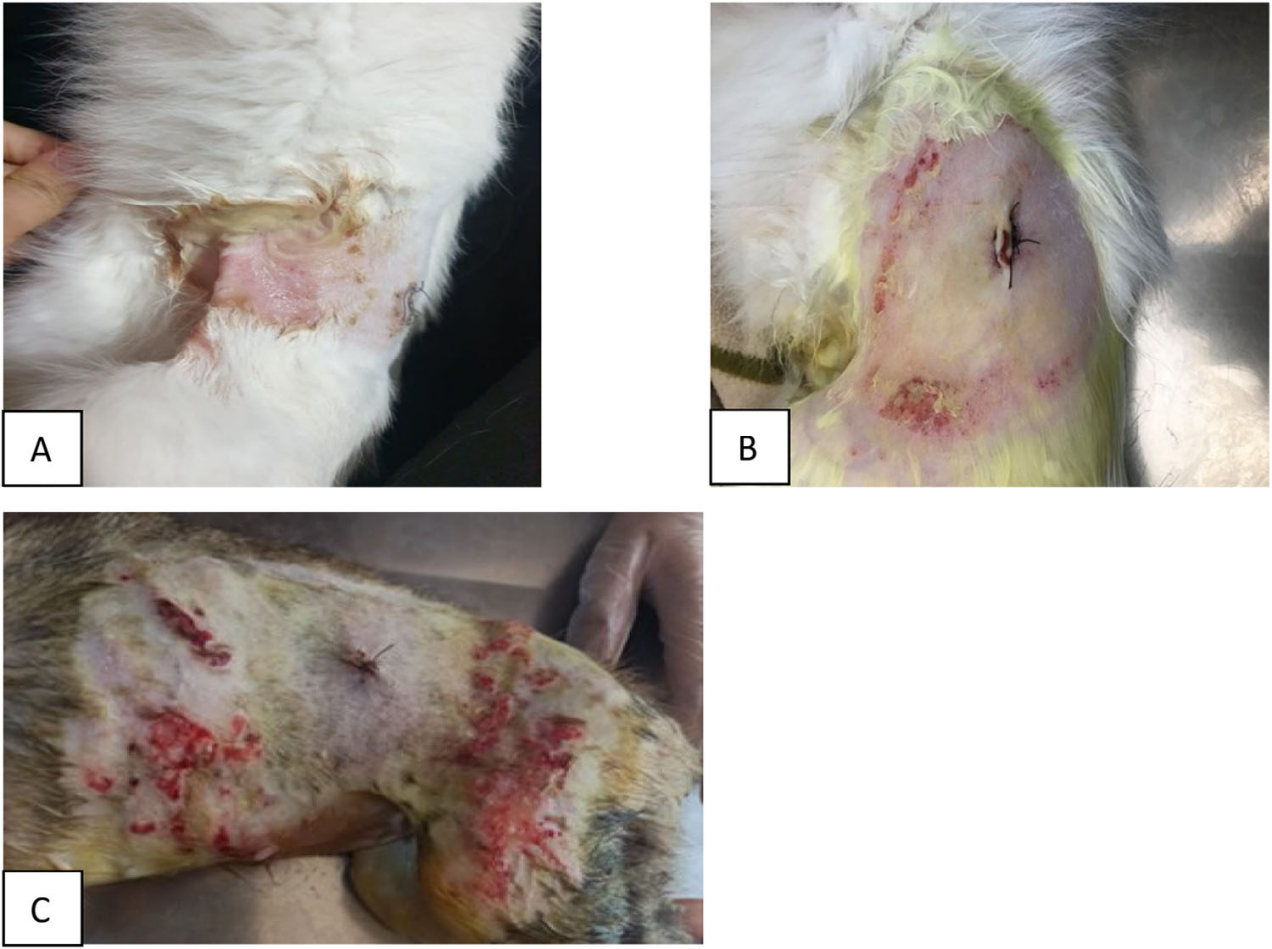
Skin problems in cats with FCV infection.

In treatment, broad‐spectrum antibiotics against secondary infections (Amoxicillin–clavulanic acid, Synulox, Pfizer 12.5 mg/kg), glucocorticoid (Dexamethasone 0.2 mg/kg), nonsteroidal anti‐inflammatory (Meloxicam, Bavet meloxicam, Bavet 0.2 mg/kg), vitamin E (Evicap, 30 IU/kg), dexpanthenol (Bepanthene, 11 mg/kg) and vitamin C (Zinco‐C, 150 mg/cat) were administered. A topical solution of glycerin iodine was applied to the oral lesions on the tongue. In addition, fluid therapy supplement was applied by calculating daily needs and fluid losses in animals with stagnation and loss of appetite. Wound treatment was performed with daily wet bandage application with ethacridine lactate (Rivanolum powder) in patients with ulcerative dermatitis (Figure 2). The healing process took about 3–10 days, depending on the complications observed.

### 2.2 Statistical Analysis

Statistical analyses were performed with the use of IBM SPSS Statistics 27.0 software (IBM Corp., USA). Fisher's exact test was used to determine whether there were differences in the occurrence of clinical symptoms, breed distribution, presence of skin lesions or healing time between the vaccinated and non‐vaccinated groups. For all statistical analyses, a *p* value less than 0.05 was considered statistically significant.

## 3 Results

There was no significant difference in the occurrence of clinical symptoms between vaccinated and non‐vaccinated cats. This suggests that vaccination did not prevent or reduce symptom occurrence in affected cats. There was a significant difference in breed distribution between vaccinated and non‐vaccinated groups (*p* < 0.05). This indicates that certain breeds were more likely to be vaccinated, potentially influencing the dataset's demographic structure. There was no significant difference in symptom onset time (*p* = 0.822), presence of skin lesions (*p* = 1.000) and healing time (*p* = 0.235) between vaccinated and non‐vaccinated cats. The statistical analysis in this study was limited by the small sample size, which reduced the power to detect significant associations and precluded the use of more complex statistical modelling. Fisher's exact test was selected as the most appropriate method for evaluating associations between categorical variables in small datasets. However, the results should be interpreted with caution due to the limited sample size and the exploratory nature of the study. Future studies with larger cohorts will be essential to validate these findings and to allow for more comprehensive statistical approaches.

## 4 Discussion

FCV infection can cause a typical tongue ulceration and upper respiratory signs but has also been associated with chronic stomatitis, which may be immune‐mediated. Clinical findings after FCV infection differ depending on the virulence of the infecting strain, the age of the affected cat and husbandry factors (Pedersen et al. 2000; Schorr‐Evans et al. 2003; Coyne et al. 2006; Park et al. 2024). Although other publications have reported tongue ulceration rates between 12.5% and 46%, in our cases the incidence of oral ulceration was 100% as reported by Deschamps et al. (2015) (Schorr‐Evans et al. 2003; Park et al. 2024). The average incubation period was reported to be 4.5 days, which aligns with other publications; however, in our case, it was observed as 2 days (Deschamps et al. 2015). Due to its environmental stability and high mutation rate, FCV poses a persistent challenge in veterinary settings, particularly in multi‐cat environments such as shelters, catteries and veterinary hospitals (Schorr‐Evans et al. 2003; Park et al. 2024). While there is no direct literature linking OVH procedures to nosocomial FCV outbreaks, the stress associated with surgery and hospitalization could highly predispose cats to viral infections or exacerbate existing subclinical conditions. Declercq (2005) described two cases of pustular dermatitis caused by FCV after ovariectomy. Similarly, skin lesions were seen in seven of our cases, and on average, they started on the third day. The occurrence of typical ulceration areas on the tongue and increased salivation, followed by skin problems in the subsequent days, ruled out the possibility of bacterial dermatitis in our cases. Declercq (2005) was reported that it was unclear whether the cats were infected during surgery, in one case, clinical diagnosis couldn't be made due to delayed observation of lesions on the tongue, and resulting in the cat's death. On the other hand, virulent systemic FCV disease is characterized with mortality rates of up to 79% (Hurley et al. 2004; Park et al. 2024). Therefore, stringent infection control measures are essential during surgical procedures to minimize the risk of nosocomial infections. This includes proper sterilization of surgical instruments, maintaining a clean environment and isolating infected or susceptible animals. While the incubation period is 1–5 days in patients with virulent systemic FCV infection in hospitals, it can be extended up to 12 days in the home environment (Hurley and Sykes. 2003; Declercq 2005).

The diagnosis of FCV is multifaceted, utilizing viral isolation, molecular techniques such as reverse transcription polymerase chain reaction (RT‐PCR), and serological assays to confirm the presence of the virus and assess its impact on feline health. Each method has its advantages and limitations, and often, a combination of these techniques is employed to ensure accurate diagnosis and effective management of FCV infections in cats (Abd‐Eldaim et al. 2009; Radford et al. 2009; Bordicchia et al. 2021). Although swab and blood samples were collected, molecular diagnostic methods such as RT‐PCR or virus isolation could not be performed, as this was not a planned or funded research project. Despite the absence of external financial support, we acknowledge the critical importance of molecular confirmation for definitive diagnosis. For this reason, suspected FCV diagnosis were clarified by performing FCV antibody titre measurements in our cases. Even though molecular investigation was used false‐negative results can occur for several reasons, including the quality of the collected samples, the conditions under which the samples are transported, and the appropriateness of the primers used in molecular diagnostics (Radford et al. 2007; Acar and Bilge‐Dagalp 2025). The high genetic diversity of calicivirus can also contribute to these issues (Acar and Bilge‐Dagalp 2025). No matter which diagnostic method is employed for detecting FCV infection, the interpretation of test results should always be guided by the presence of corresponding clinical signs (Hurley and Sykes 2003; Deschamps et al. 2015). Although it has been reported that the detection of antibodies as a serological analysis does not fully confirm active infection, interpreting it together with clinical symptoms and initiating rapid treatment yielded positive results in our reports. Moreover, in patients showing systemic signs associated with suspected FCV infection, oral and lingual lesions may appear at different stages of the disease; therefore, repeated daily examinations of the tongue and oral cavity may be necessary (Declercq 2005).

Vaccination against FCV has been available for many years and has effectively reduced the incidence of clinical disease. However, evidence from the field suggests that the current vaccines do not prevent FCV associated VSD with outbreaks occurring in vaccinated cats (Coyne et al. 2006; Hurley et al. 2004). Likewise, we know that some of the 7 cats in our cases were vaccinated. On the other hand, regional variations in awareness regarding feline vaccination were observed. Specifically, in the city where this outbreak occurred, the vaccination rate was relatively low, while the practice of keeping stray cats was notably common. FCV has a high level and rapid mutation ability, and we think that vaccines cannot provide adequate protection against different biotypes in cats due to these features.

The molecular investigation of FCV in Turkey is still developing, with limited studies contributing to a better understanding of its epidemiology and genetic diversity (Dokuzeylul et al. 2016; Dağalp et al. 2019; Acar and Bilge‐Dagalp 2025). Future research should aim to standardize methodologies across studies to facilitate comparative analyses and enhance our understanding of FCV dynamics in the region.

The treatment options for FCV are limited, primarily due to the absence of specific antiviral therapies approved for use in cats. The practical application of antiviral agents in clinical settings requires further validation through controlled studies (Fumian et al. 2018; Bordicchia et al. 2021). Current management strategies focus on supportive care, symptomatic treatment and the use of certain investigational antiviral agents. Supportive care is crucial for cats infected with FCV, especially those exhibiting severe clinical signs such as oral ulcerations, respiratory distress or systemic illness. This includes ensuring adequate hydration, nutritional support and pain management. In cases of severe gingivostomatitis, analgesics and anti‐inflammatory medications may be administered to alleviate discomfort (Declercq 2005; Radford et al. 2009; Hermawan and Leo 2022). In addition, secondary bacterial infections are common in FCV‐infected cats, necessitating the use of antibiotics to manage these complications (Declercq 2005). The use of systemic glucocorticoids in the treatment of FCV infections is a topic of interest due to the immunomodulatory effects of glucocorticoids and their potential role in managing inflammatory responses associated with viral infections. While glucocorticoids are not antiviral agents, they can be beneficial in alleviating symptoms and managing complications arising from FCV infections, particularly in cases of severe oral lesions or secondary bacterial infections. Glucocorticoids are known to exert significant immunosuppressive effects, which can be beneficial in cases where an overactive immune response contributes to tissue damage. In FCV‐infected cats, particularly those suffering from chronic gingivostomatitis, glucocorticoids may help reduce inflammation and pain associated with oral lesions (Declercq 2005; Wei et al. 2024). The use of glucocorticoids in such cases is aimed at improving the quality of life for affected cats by alleviating discomfort and promoting healing. While direct studies on the use of glucocorticoids specifically for FCV are limited, studies have shown that glucocorticoids can help manage severe inflammatory responses in other viral infections, suggesting a similar approach could be beneficial for FCV (Declercq 2005). Based on the treatment results, cats began to show an increased appetite approximately 1–2 days after the application of glucocorticoids, which helped reduce inflammation on their tongues. Consequently, all cats showed an overall improvement in their condition, and there were no fatal cases reported. On the other hand, delayed wound healing is reported as a potential risk associated with glucocorticoid therapy. Although there were patients with skin problems due to suspected FCV, none of them experienced any problems in the operation area. However, their use should be approached with caution, considering the potential risks and the specific clinical context of each case. In addition to regular application of wet bandages to patients with skin problems, vitamins E, C and dexpanthenol were used. The use of vitamins E, C and dexpanthenol, has been widely studied for their beneficial effects on skin health, particularly in promoting wound healing, and providing anti‐inflammatory properties (Heise et al. 2012; Karakaya‐Bilen and Akgün 2021). For this reason these were added to the treatment protocol specifically for patients with skin problems.

This study presents the first report of clustered cases suggestive of FCV infection in eastern Turkey, observed on three separate occasions in a veterinary training and research hospital. Although some clinical signs observed in this outbreak—such as oral ulceration, lethargy, hypersalivation and skin lesion—may overlap with those reported in virulent systemic FCV (VSD‐FCV) cases, our findings do not fully meet the established criteria for confirmed VSD. Specifically, all affected cats recovered with supportive care, and there was no evidence of multisystemic organ failure or high mortality, which are considered hallmark features of VSD‐FCV. Furthermore, due to the absence of molecular testing such as RT‐PCR or viral sequencing, definitive identification of a VSD strain was not possible. Therefore, the clinical presentation observed in this study is more appropriately interpreted as a suspected systemic manifestation of FCV infection rather than a confirmed VSD‐FCV outbreak.

There are some limitations of this study, one is the absence of a control group consisting of cats that underwent ovariohysterectomy during the same period but did not develop clinical signs. As no systematic follow‐up or surveillance data were available for asymptomatic cases, it remains uncertain whether the infections observed were purely coincidental or represented a true nosocomial outbreak. However, it is worth noting that previous reports of suspected FCV outbreaks in veterinary settings have also typically lacked formal control groups, particularly when the cases emerged unexpectedly and were documented retrospectively (Reynolds et al. 2009; Deschamps et al. 2015; Park et al. 2024). Another important limitation of this study is the lack of a detailed epidemiological investigation, including environmental sampling and assessment of potential fomite transmission routes. These methods could have provided stronger evidence supporting a nosocomial origin of the outbreak. However, due to the retrospective and unplanned nature of the event, no such procedures could be implemented at the time. Immediate efforts were instead directed toward clinical management, rapid disinfection of surgical areas and temporary hospital closure to contain further spread. Despite the absence of confirmatory environmental data, the temporal clustering of clinical signs shortly after ovariohysterectomy in unrelated cats, together with their recovery following similar therapeutic protocols, supports the possibility of a hospital‐associated source. Nevertheless, the findings must be interpreted with caution, and future outbreak investigations should incorporate systematic environmental surveillance and epidemiological tracking.

## 5 Conclusion

FCV is highly prevalent and poses problems mainly in multicat environments and when hygienic conditions are suboptimal. Given the potential severity and rapid spread of FCV‐VSD, early recognition and prompt implementation of biosecurity measures are critical in controlling outbreaks in veterinary settings. In this study, lesions observed in the postoperative period in cats that underwent ovariohysterectomy were evaluated in the context of suspected FCV infection In conclusion, to the best of the authors’ knowledge, this is the first report describing the clinical presentation, presumptive diagnosis and treatment outcomes of suspected FCV infections occurring in cats following ovariohysterectomy procedures at different times in a veterinary training and research hospital. During this process, it is important to close hospitals and ensure disease control through comprehensive disinfection and to always pay attention to biosecurity.

## Author Contributions

**Ebru Karakaya‐Bilen**: investigation, writing – original draft, data curation, methodology, validation, writing – review and editing, visualization. **Gülşah Akgül**: investigation, methodology, validation, writing ‐ review and editing. **Oznur Yılmaz‐Koc**: data curation, investigation, validation, methodology, visualization, writing – review and editing.

## Ethics Statement

The authors confirm that the ethical policies of the journal, as noted on the journal's author guidelines page, have been adhered to. No ethical approval was required as this is a review article with no original research data.

## Conflicts of Interest

The authors declare no conflicts of interest.

## Peer Review

The peer review history for this article is available at https://publons.com/publon/10.1002/vms3.70540.

## Data Availability

The authors have nothing to report.

## References

[vms370540-bib-0001] Abd‐Eldaim, M. , R. P. Wilkes , K. V. Thomas , and M. Kennedy . 2009. “Development and Validation of a Taqman Real‐Time Reverse Transcription‐PCR for Rapid Detection of Feline Calicivirus.” Archives of Virology 154, no. 4: 555–560. 10.1007/s00705-009-0337-5. 19253013 PMC7086925

[vms370540-bib-0002] Acar, G. , and S. Bilge‐Dagalp . 2025. “Investigation of the Epidemiology of Calicivirus Infection of Cats Using Molecular and Virus Isolation Techniques.” Comparative Immunology, Microbiology and Infectious Diseases 119: 102335. 10.1016/j.cimid.2024.102335. 40081119

[vms370540-bib-0003] Bordicchia, M. , T. M. Fumian , K. Van Brussel , et al. 2021. “Feline Calicivirus Virulent Systemic Disease: Clinical Epidemiology, Analysis of Viral Isolates and In Vitro Efficacy of Novel Antivirals in Australian Outbreaks.” Viruses 13: 2040. 34696470 10.3390/v13102040 PMC8537534

[vms370540-bib-0004] Caringella, F. , G. Elia , N. Decaro , et al. 2019. “Feline Calicivirus Infection in Cats With Virulent Systemic Disease.” Italy Research in Veterinary Science 124: 46–51. 10.1016/j.rvsc.2019.02.008. 30844542

[vms370540-bib-0005] Coyne, K. P. , B. R. Jones , A. Kipar , et al. 2006. “Lethal Outbreak of Disease Associated With Feline Calicivirus Infection in Cats.” Veterinary Record 158: 544–550. 16632527 10.1136/vr.158.16.544

[vms370540-bib-0006] Dağalp, B. S. , F. Doğan , T. A. Farzani , et al. 2019. “Molecular Investigation of Feline Herpesvirus 1 (FHV‐1) and Feline Calicivirus in Cats With Respiratory System Problem.” Eurasian Journal of Veterinary Science 35, no. 3: 131–138.

[vms370540-bib-0007] Declercq, J. 2005. “Pustular Calicivirus Dermatitis on the Abdomen of Two Cats Following Routine Ovariectomy.” Veterinary Dermatology 16, no. 6: 395–400. 10.1111/j.1365-3164.2005.00475.x. 16359307

[vms370540-bib-0008] Deschamps, J. Y. , E. Topie , and F. Roux . 2015. “Nosocomial Feline Calicivirus‐Associated Virulent Systemic Disease in a Veterinary Emergency and Critical Care Unit in France.” Journal of Feline Medicine and Surgery Open Reports 1, no. 2: 2055116915621581. 10.1177/2055116915621581. 28491401 PMC5362001

[vms370540-bib-0009] Dokuzeylul, B. , A. Kayarm , and M. E. Or . 2016. “Prevalence of Systemic Disorders in Cats With Oral Lesions.” Veterinarni Medicina 61, no. 4: 219–223.

[vms370540-bib-0010] Gaskell, R. M. , S. Dawson , and A. Radford . 2006. “Feline Respiratory Disease.” In Infectious Diseases of the Dog and Cat, edited by G. E. Greene , 145–154. Saunders Elsevier.

[vms370540-bib-0011] Heise, R. , C. Skazik , Y. Marquardt , et al. 2012. “Dexpanthenol Modulates Gene Expression in Skin Wound Healing In Vivo.” Skin Pharmacology and Physiology 25, no. 5: 241–248. 10.1159/000341144. 22759998

[vms370540-bib-0012] Hermawan, I. P. , and D. M. Leo . 2022. “Feline Calicivirus Infection With Chronic Stomatitis, Rhinitis and Otitis in a Bengal Cat in Indonesia.” Veterinary Biomedical and Clinical Journal 4, no. 2: 46–50. 10.21776/ub.vetbioclinj.2022.004.02.1.

[vms370540-bib-0013] Hofmann‐Lehmann, R. , M. J. Hosie , K. Hartmann , et al. 2022. “Calicivirus Infection in Cats.” Viruses 14, no. 5: 937–968. 35632680 10.3390/v14050937 PMC9145992

[vms370540-bib-0014] Hurley, K. F. , P. A. Pesavento , N. C. Pedersen , et al. 2004. “An Outbreak of Virulent Systemic Feline Calicivirus Disease.” Journal of the American Veterinary Medical Association 224, no. 2: 241–249. 10.2460/javma.2004.224.241. 14736069

[vms370540-bib-0015] Hurley, K. F. , and J. E. Sykes . 2003. “Update on Feline Calicivirus: New Trends.” Veterinary Clinics of North America Small Animal Practice 33: 759–772. 12910742 10.1016/s0195-5616(03)00025-1

[vms370540-bib-0016] Fumian, T. M. , D. E. Tuipulotu , N. E. Netzler , et al. 2018. “Potential Therapeutic Agents for Feline Calicivirus Infection.” Viruses 10, no. 8: 433. 10.3390/v10080433. 30115859 PMC6116245

[vms370540-bib-0017] Karakaya Bilen, E. , and M. B. Akgül . 2021. “Acquired Skin Fragility Syndrome in a Juvenile Cat Following a Routine Ovariohysterectomy.” Acta Scientiae Veterinariae 49. 10.22456/1679-9216.105305.

[vms370540-bib-0018] Kreisler, R. E. , S. L. Shaver , and J. H. Holmes . 2018. “Outcomes of Elective Gonadectomy Procedures Performed on Dogs and Cats by Veterinary Students and Shelter Veterinarians in a Shelter Environment.” Journal of the American Veterinary Medical Association 253, no. 10: 1294–1299. 30398427 10.2460/javma.253.10.1294

[vms370540-bib-0019] Park, J. , D. Lee , Y. Hong , C. Hwang , and J. Hyun . 2024. “Outbreaks of Nosocomial Feline Calicivirus‐Associated Virulent Systemic Disease in Korea.” Journal of Veterinary Science 25, no. 4: e51. 10.4142/jvs.24030. 39083203 PMC11291428

[vms370540-bib-0020] Pedersen, N. C. , J. B. Elliott , A. Glasgow , et al. 2000. “An Isolated Epizootic of Hemorrhagic‐Like Fever in Cats Caused by a Novel and Highly Virulent Strain of Feline Calicivirus.” Veterinary Microbiology 73: 281–300. 10781727 10.1016/S0378-1135(00)00183-8 PMC7117377

[vms370540-bib-0021] Radford, A. D. , K. P. Coyne , S. Dawson , et al. 2007. “Feline Calicivirus.” Veterinary Research 38, no. 2: 319–335. 10.1051/vetres:2006056. 17296159

[vms370540-bib-0022] Radford, A. D. , D. Addie , S. Belák , et al. 2009. “Feline Calicivirus Infection: ABCD Guidelines on Prevention and Management.” Journal of Feline Medicine and Surgery 11, no. 7: 556–564. 19481035 10.1016/j.jfms.2009.05.004 PMC11132273

[vms370540-bib-0023] Reynolds, B. S. , H. Poulet , J. L. Pingret , et al. 2009. “A Nosocomial Outbreak of Feline Calicivirus Associated Virulent Systemic Disease in France.” Journal of Feline Medicine and Surgery 11, no. 8: 633–644. 10.1016/j.jfms.2008.12.005. 19201637 PMC11132575

[vms370540-bib-0024] Roberts, M. L. , J. A. Beatty , N. K. Dhand , and V. R. Barrs . 2015. “Effect of Age and Surgical Approach on Perioperative Wound Complication Following Ovariohysterectomy in Shelter‐Housed Cats in Australia.” Journal of Feline Medicine and Surgery Open Reports 1, no. 2: 2055116915613358. 28491391 10.1177/2055116915613358 PMC5362017

[vms370540-bib-0025] Schorr‐Evans, E. M. , A. Poland , W. E. Johnson , and N. C. Pedersen . 2003. “An Epizootic of Highly Virulent Feline Calicivirus Disease in a Hospital Setting in New England.” Journal of Feline Medicine and Surgery 5, no. 4: 217–226. 10.1016/S1098-612X(03)00037-7. 12878149 PMC10822567

[vms370540-bib-0026] Wei, Y. , Q. Zeng , H. Gou , and S. Bao . 2024. “Update on Feline Calicivirus: Viral Evolution, Pathogenesis, Epidemiology, Prevention and Control.” Frontiers in Microbiology 15: 1388420. 10.3389/fmicb.2024.1388420. 38756726 PMC11096512

